# Blockade of soluble epoxide hydrolase attenuates post-ischemic neuronal hyperexcitation and confers resilience against stroke with TrkB activation

**DOI:** 10.1038/s41598-017-18558-6

**Published:** 2018-01-08

**Authors:** Li-Hsin Chang, Hui-Ching Lin, Shiang-Suo Huang, I-Chih Chen, Kai-Wen Chu, Chun-Lien Chih, Yao-Wen Liang, Yi-Chung Lee, You-Yin Chen, Yi-Hsuan Lee, I-Hui Lee

**Affiliations:** 1 Institute of Brain Science, National Yang-Ming University, Taipei, Taiwan; 2 Department and Institute of Physiology, National Yang-Ming University, Taipei, Taiwan; 3 Department of Pharmacology, Institute of Medicine, Chung-Shan Medical University, Taichung, Taiwan; 4 Cheng Hsin General Hospital, Taipei, Taiwan; 5 Department of Life Sciences and Institute of Genome Sciences, National Yang-Ming University, Taipei, Taiwan, Taipei, Taiwan; 6 Department of Neurology, Taipei Veterans General Hospital, Taipei, Taiwan; 7 Department of Neurology, School of Medicine National Yang-Ming University, Taipei, Taiwan; 8 Department of BioMedical Engineering, National Yang-Ming University, Taipei, Taiwan

## Abstract

Inhibition and deletion of soluble epoxide hydrolase (sEH) has been suggested to ameliorate infarction in experimental ischemic stroke possibly via vasoactive epoxyeicosatrienoic acids. However, it is unknown whether the neuroprotective mechanisms involve alteration of post-ischemic neuronal transmission and neurotrophic signaling. We used a permanent middle cerebral artery occlusion (MCAO) model in adult wild-type mice with the sEH inhibitor 12-(3-adamantan-1-yl-ureido)dodecanoic acid (AUDA) post-treatment and in sEH knockout (sEH KO) mice. We found that sensorimotor recovery was significantly enhanced after MCAO in both AUDA-treated and sEH KO mice, with decreased sEH activity and brain infarction. Decreased post-ischemic long-term potentiation (iLTP) was observed in an *ex vivo* hippocampal oxygen-glucose deprivation model. Tropomyosin receptor kinase B (TrkB) activation, rather than glutamate receptor alteration, was consistently found after the different manipulations. Immunohistochemistry further revealed peri-infarct neuronal TrkB activation and microvasculature augmentation in AUDA-treated and sEH KO mice, suggesting parallel neurovascular enhancement. Mechanistically, pretreatment with a selective TrkB antagonist ANA12 countered the effect of iLTP attenuation induced by sEH deletion *ex vivo* and abolished the infarct reduction *in vivo*. Together, the neuroprotective effects of sEH inhibition and gene deletion can both be mediated partially via enhancement of TrkB signaling which attenuated post-ischemic neuroexcitation and neurological deficits.

## Introduction

Soluble epoxide hydrolase (sEH), which is encoded by the EPHX2 gene, is found in nearly all mammalian tissues^1^ as an enzyme with dual activities, including the C-terminal hydrolase activity responsible for the metabolism of epoxyeicosatrienoic acids (EETs) into less-active dihydroxyeicosatrienoic acids (DHETs)^2^, and the N-terminal phosphatase activity^3^. EETs and related epoxide fatty acids are signaling fatty acids generated from arachidonic acid through the cytochrome P450 epoxygenase pathway and have been shown to have protective effects on vasodilation^4,5^, angiogenesis^6^, as well as anti-inflammation^7–10^. It has been shown that sEH expression in the mammalian brain occurs mainly in astrocytes and vascular endothelial and smooth muscle cells as well as in a few neuronal populations in the striatum, cortex, and hippocampus^11–14^. Following middle cerebral artery occlusion (MCAO) in adult rodents, cytochrome P450 enzymes and arachidonic acid metabolites have been suggested to play essential roles in regulating cerebral blood flow and inflammatory responses to ischemia. Systemic administration of 12-(3-adamantan-1-yl-ureido)dodecanoic acid (AUDA), an sEH inhibitor, has been shown to reduce infarct size and ameliorate functional deficits; these effects were attributed, at least partially, to increased EET levels and enhanced cerebral blood flow^11,15,16^. Similarly, sEH knockout (sEH KO) mice were also protected from ischemic stroke. Compared to wild-type (WT) controls, sEH KO mice had smaller infarcts in association with increased EET levels and collateral blood flow^11,12^, In contrast, transgenic mice with endothelial-specific overexpression of human sEH exhibited impaired vasodilatation and enlarged infarcts after MCAO in female but not in male mice^17^, suggesting that sEH may serve as a vasoactive modulator and a potential therapeutic target in ischemic stroke. However, the functional relevance of neuroglial sEH expression, as well as sEH blockade-mediated neuroprotective mechanisms against stroke, remain elusive.

A recent study reported that sEH KO mice subjected to MCAO had decreased infarcts in association with elevated brain-derived neurotrophic factor (BDNF) expression predominantly in peri-infarct glial fibrillary acidic protein (GFAP)-positive astrocytes^18^. The infarct reduction was countered by intracerebroventricular injection of a potent but non-selective tyrosine receptor kinase inhibitor, K252a, which acts on tropomyosin receptor kinase (Trk)A, TrkB or TrkC. Moreover, exogenous 14,15-EET treatment in cultured murine astrocytes and a human astroglioma cell line subjected to oxygen-glucose deprivation (OGD) increased BDNF expression and cell viability, indicating that 14,15-EET-mediated production of BDNF by astrocytes and this effect might protect against ischemic injury in sEH KO mice^18^. In another study that used lipopolysaccharide-induced inflammation and repeated social defeat stress models, pretreatment with an sEH inhibitor and sEH KO were found to prevent the onset of depression-like behaviors and to increase BDNF-TrkB expression in the prefrontal cortex and the hippocampus, suggesting that BDNF-TrkB signaling confers the stress resilience in association with sEH blockade^19^. Nevertheless, these interesting findings have not elucidated how sEH blockade alters neural excitability after ischemic injury and whether BDNF-TrkB signaling mediates such neuroprotective effects. BDNF is a paracrine neurotrophic factor^20^ that is secreted by almost all cell types in the brain. BDNF binds to the high-affinity transmembrane receptor TrkB and the low-affinity p75 neurotrophin receptor to mediate various neuroplastic and neuroprotective effects throughout life^21,22^. Here, we investigate how pharmacological inhibition and gene deletion of sEH exerts neuroexcitatory modulation and neuroprotection against ischemic hypoxic brain injury by utilizing N-[2-[[(Hexahydro-2-oxo-1H-azepin-3-yl) amino] carbonyl] phenyl]-benzo[b] thiophene-2-carboxamide (ANA12), a selective TrkB inhibitor^23^, and focusing on the role of BDNF-TrkB signaling and glutamate receptors. To do so, we adopted a permanent mouse MCAO model *in vivo* and post-ischemic long-term potentiation (iLTP) measurements in *ex vivo* hippocampal slices subjected to OGD. Given the potential neurovascular benefits of sEH inhibitors in preclinical stroke models and their safety profiles in humans^24,25^, our findings indicate that TrkB activation is involved in sEH blockade-mediated neuroprotection in acute ischemic stroke and open new possibilities for combination treatments with neuroprotectants and reperfusion by thrombolytic and/or endovascular thrombectomy treatments.

## Results

### Both pharmacological inhibition and gene deletion of sEH attenuate acute cerebral infarction, ischemia-induced sEH activity, and sensorimotor deficits

**Figure 1:**
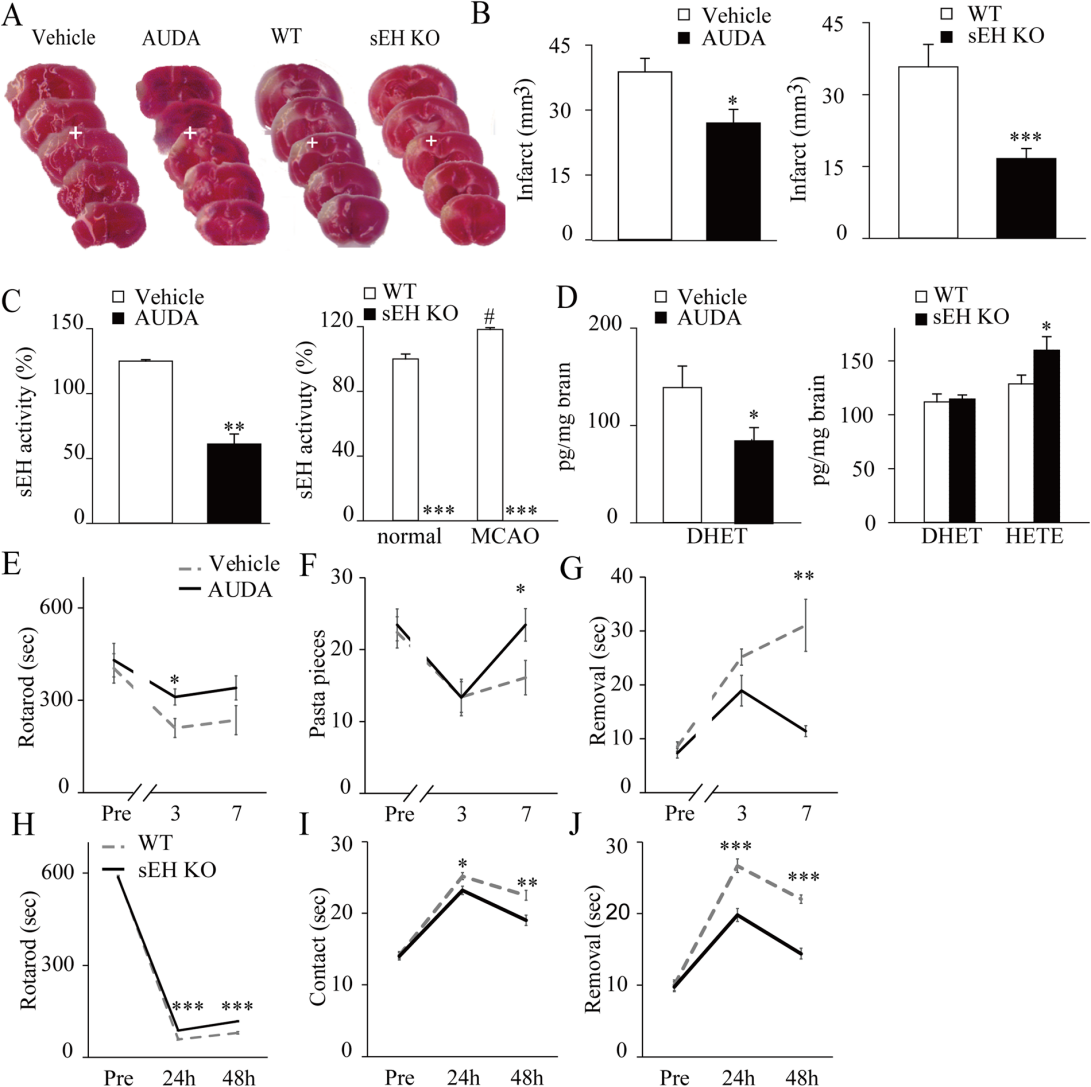
Pharmacological inhibition and gene deletion of soluble epoxide hydrolase (sEH) attenuate acute cerebral infarction, sEH activity, and sensorimotor deficits. (**A**) Representative brain slices from vehicle- vs. sEH inhibitor (AUDA)-treated C57BL/6 mice that were stained with vital dye, and from wild-type (WT) vs. sEH knockout (sEH KO) mice at 48 hours after middle cerebral artery occlusion (MCAO) (the white crosses indicate the peri-infarct cortices on which the immunohistochemistry was shown in Fig. 4). (**B**) The infarct volume was reduced in the AUDA-treated group (n = 18) compared to the vehicle-treated group (n = 17). Similarly, the sEH KO group (n = 8) had significantly smaller infarcts than the WT group (n = 8). (**C**) The average sEH hydrolase activity in the ipsilesional hemisphere (represented as fold changes relative to the normal WT baseline) increased after MCAO in the control vehicle and WT groups (n = 5/group). AUDA treatment reduced sEH activity, and there was no sEH activity in the sEH KO mice relative to the corresponding controls (n = 5/group). (**D**) The mean level of 14,15-DHET in the ipsilesional hemisphere decreased with AUDA treatment relative to that observed following vehicle treatment but did not differ between the WT and sEH KO groups. However, the mean level of 20-HETE was elevated in the sEH KO group compared to the WT group (n = 5/group). (**E**) The performance of the AUDA-treated mice (n = 9) was better than that of the vehicle-treated controls (n = 10) on the rotarod test, (**F**) the pasta matrix reaching task and (**G**) the adhesive removal test by 7 days after MCAO. **(H**) Likewise, the sEH KO mice (n = 5) exhibited reduced sensorimotor deficits compared to the WT mice (n = 6) during the rotarod test, (**I**) reduced adhesive contact and (**J**) reduced adhesive removal time by 48 hours after MCAO. The results are expressed as the mean ± standard error of the mean. *p < 0.05, **p < 0.01, ***p < 0.001 compared with controls.

Consistent with previous studies^15,26^, we found that both the AUDA-treated mice and soluble epoxide hydrolase knockout (sEH KO) mice exhibited a significant reduction of infarct volume relative to the vehicle-treated and wild type (WT) mice (Fig. 1A,B). Acute cerebral ischemia increased sEH activity in WT mice. AUDA treatment decreased sEH activity relative to vehicle treatment, while no sEH activity was detected in the brains of sEH KO mice either before or after middle cerebral artery occlusion (MCAO) (Fig. 1C). Cytochrome P450 (CYP) epoxygenases and hydroxylases metabolize arachidonic acid into epoxyeicosatrienoic acids (EETs) (CYP2B, CYP2C, and CYP2J isoforms) and hydroxyeicosatetraenoic acids (HETEs) (CYP4A and CYP4F isoforms), respectively. EETs are degraded by sEH into the corresponding less-active dihydroxyeicosatrienoic acids (DHETs). Thus, we measured 14,15-DHET levels as surrogates for EET-to-DHET ratios and found that sEH inhibition decreased 14,15-DHET levels relative to those found in controls; however, sEH KO had no impact (Fig. 1D). By contrast, sEH KO significantly increased the 20-HETE levels relative to those in controls (Fig. 1D), which is in line with others’ findings. These findings suggest that reduced EET degradation in sEH KO mice might have shifted the cytochrome P450 monooxygenase metabolism from epoxy-arachidonic acid derivatives to more hydroxyl-arachidonic acid derivatives^27,28^. Moreover, the AUDA-treated mice had enhanced sensorimotor coordination and increased latencies on the accelerating rotarod test (Fig. 1E). They also displayed significantly improved performance on the skilled forelimb reaching task (Fig. 1F) and the adhesive removal test at 7 days post-MCAO compared to the vehicle-treated mice (Fig. 1G). Likewise, compared to the WT controls, the sEH KO mice had significantly increased latencies in the rotarod test (Fig. 1H), and the time taken to initiate contact and completely remove the adhesive was shorter at 1 and 2 days after MCAO (Fig. 1I,J). Additionally, to elucidate possible vascular mechanisms involved in the sEH-blockade neuroprotection, we longitudinally measured the tail blood pressure as well as peri-infarct cortical blood flow before and after MCAO in a separate experiment (see Supplementary methods). We found that the AUDA-treated group had a significant decrease in mean blood pressure at 24 hours after MCAO compared with the vehicle-treated and sEH KO groups (Supplementary Fig. 1A). However, the simultaneous changes of peri-infarct cortical blood flow rate in ratio to the baseline before MCAO did not show significant difference among the groups by 24 hours after MCAO (Supplementary Fig. 1B). The student t-test used in the comparisons of infarct size, sEH activity, and lipid metabolites. The repeated measures ANOVA was used to determine the behavioral changes, followed by a post-hoc with Bonferroni correction if there was a significant difference.

### Both inhibition and deletion of sEH attenuate post-ischemic hyperexcitation of Schaffer collateral-CA1 synapses in the hippocampus

**Figure 2:**
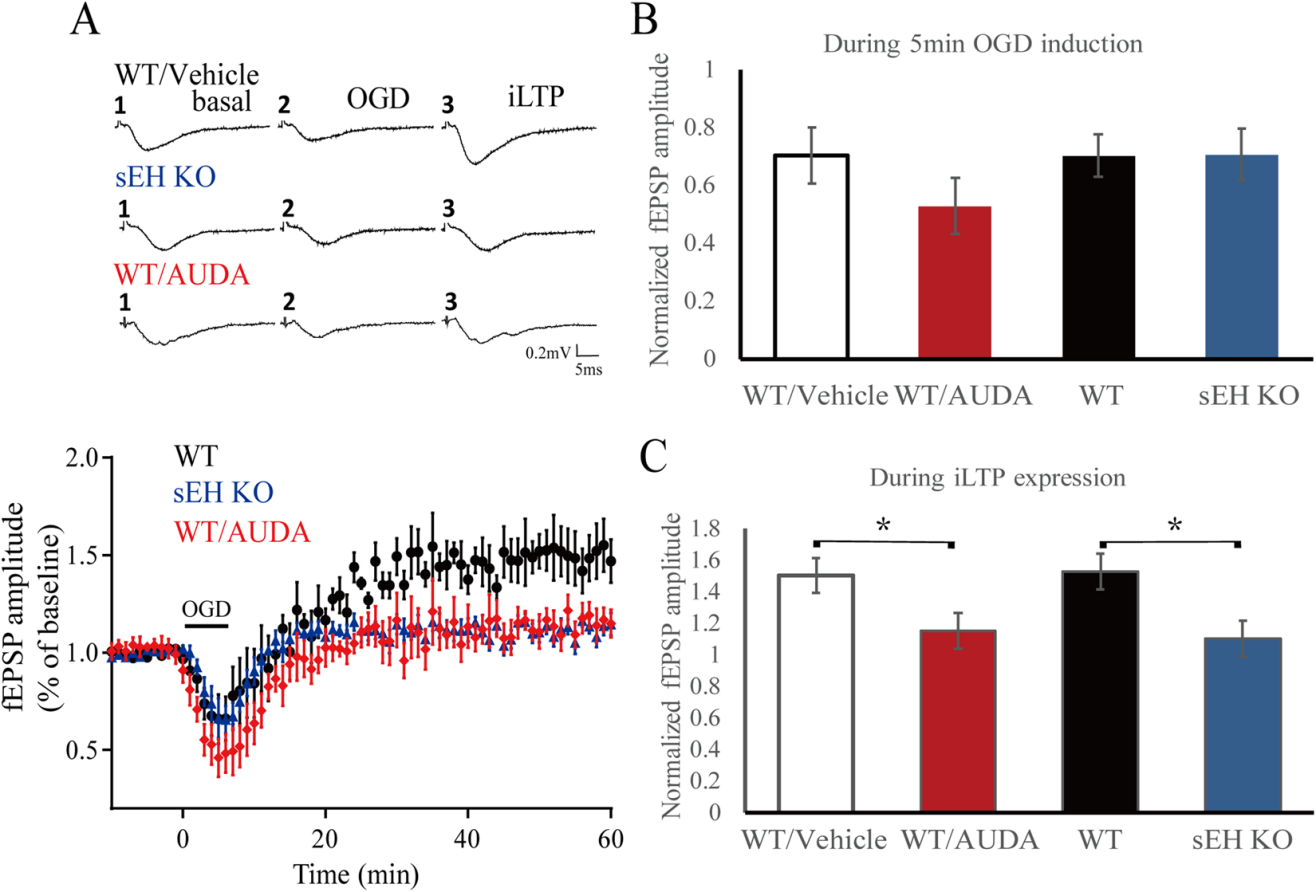
Field excitatory postsynaptic potentials (fEPSPs) evoked in the Schaffer collateral-CA1 pathway in acute hippocampal slices exposed to oxygen-glucose deprivation (OGD) for 5 minutes followed by re-oxygenation for 60 minutes. (**A**) Representative traces of fEPSPs at different time points at baseline, OGD induction, and post-ischemic long term potentiation (iLTP) expression. Averaged temporal changes in fEPSPs caused by OGD in the WT/vehicle, sEH KO and WT mice treated with AUDA (WT/AUDA). Note the presence of iLTP in the WT/vehicle mice, which characterized by the initial loss of synaptic potentials during OGD induction, and gradually recovered and even exceeded the baseline. (**B**) During the 5 minutes of OGD induction, the average amplitude of the fEPSPs did not differ among the groups (n = 6/group). (**C**) During the last 10 minutes of iLTP expression, the average fEPSP amplitude in the WT/AUDA and sEH KO groups significantly decreased relative to that in the WT/vehicle group, respectively (n = 6/group), suggesting a regulatory role of sEH on post-ischemic synaptic hyperexcitability. Calibration: 0.2 mV, 5 ms. The values represent the mean ± standard error of the mean. *p < 0.05.

Oxygen glucose deprivation (OGD)-induced post-ischemic long-term potentiation (iLTP) has been attributed to abnormal Ca^2+^ influx via the overactivation of glutamate receptors, predominantly the N-methyl-D-aspartate (NMDA) and α-amino-3-hydroxy-5-methyl-4-isoxazolepropionic acid (AMPA) receptor types^29,30^. During the 5 minutes of OGD induction, there were no obvious differences in field excitatory postsynaptic potential (fEPSP) amplitudes among the acute hippocampal slices from WT, WT/Vehicle, sEH KO and AUDA-treated (n = 6/group) mice (Fig. 2A,B), indicating the loss of synaptic potentials was similar in all groups. Interestingly, following reperfusion with oxygenated glucose solution and during the last 10 minutes of iLTP expression, the fEPSP amplitude was significantly reduced in the AUDA-treated and sEH KO slices (Fig. 2C), suggesting that sEH blockade and deletion reduced post-ischemic neuronal hyperexcitation. The student’s t-test was used to determine any statistical difference.

### Both sEH inhibition and deletion consistently upregulate post-stroke TrkB activation

To investigate how sEH is involved in postsynaptic excitability, we first examined expression of NMDA and AMPA glutamate receptors, including the NMDAR subunits GluN2A, GluN2B and the Ca^2+^-impermeable AMPAR subunit GluA2. AUDA treatment upregulated the level of the NMDAR GluN2A subunit but did not have a significant effect on GluN2B or GluA2 subunit after MCAO (Supplementary Fig. 2A). On the other hand, in sEH KO mice, GluN2B level, but not GluN2A, was significantly upregulated at normal baseline and after MCAO compared to that in the WT mice. In sEH KO mice, GluA2 level was increased at normal baseline compared that in WT controls. After MCAO, GluA2 level did not change in the sEH KO group but significantly increased in the WT controls (Supplementary Fig. 2B). Increased GluN2A subunits might trigger pro-survival signaling^31^ and contribute to neuroprotection, as well as prevent Ca^2+^ influx and thus decrease iLTP and excitotoxicity^32^. In contrast, GluN2B subunits have been suggested to be detrimental and critical for iLTP induction^33^, while GluN2A and GluN2B were inconsistently regulated by sEH pharmacological inhibition and gene deletion, which needs further investigation, other mechanisms might be involved in the sEH blockade-mediated neuroprotection.

**Figure 3:**
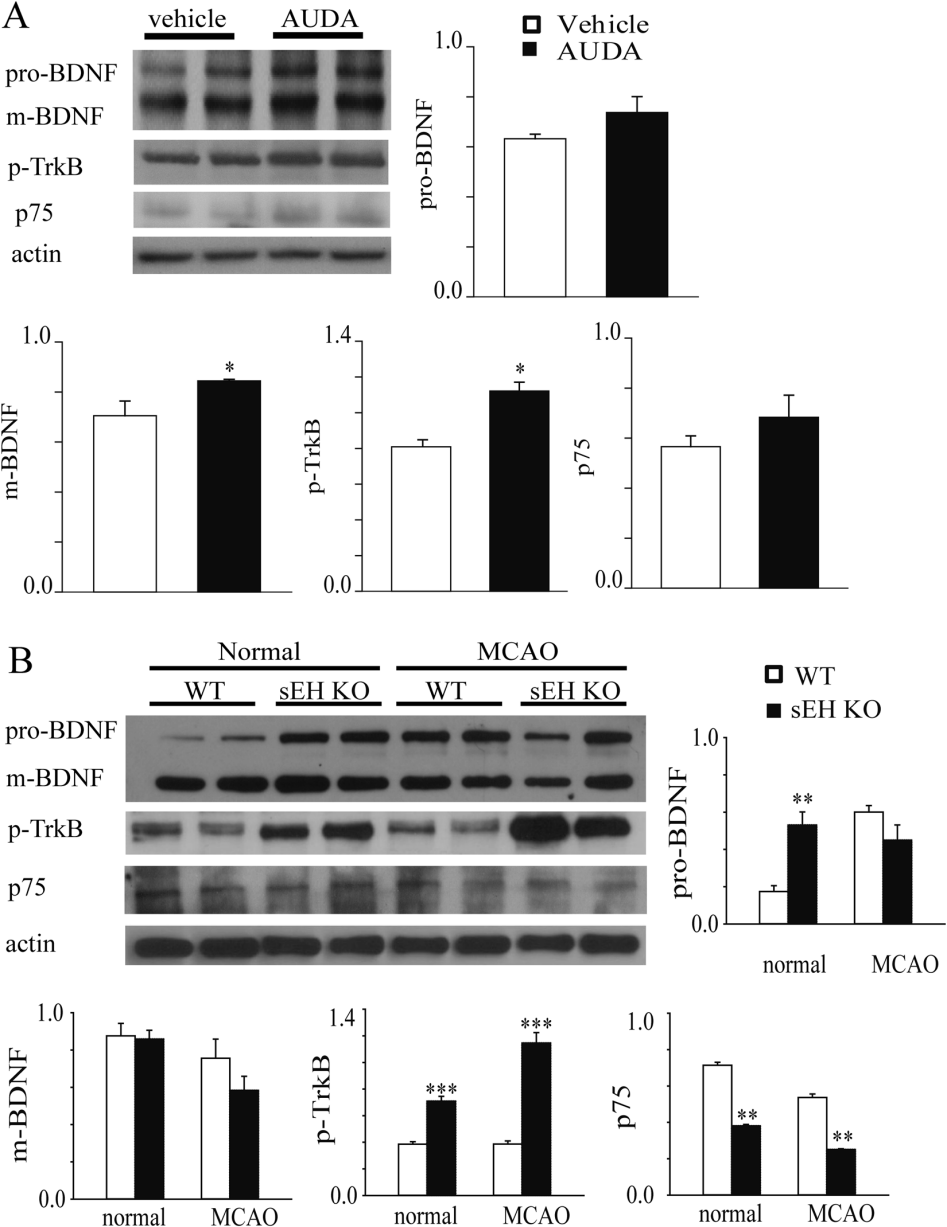
(**A**) Representative western blots of the ipsilesional hemisphere from vehicle- and AUDA-treated mice, with β-actin as the loading control. Seven days following MCAO, the AUDA treatment upregulated protein expression of mature BDNF (m-BDNF) and phosphorylated TrkB (p-TrkB). The expression levels are expressed in terms of fold changes of β-actin relative to the vehicle treatment (n = 8/group). The levels of pro-BDNF and p75 neurotrophin receptor (p75NTR) did not differ between the groups. (**B**) Representative western blots of WT and sEH KO mice before (normal) and 48 hours after MCAO, with β-actin as the loading control. The protein levels of p-TrkB in the sEH KO mice was upregulated both before and after MCAO when compared to those in the WT mice (n = 8/group). The expression levels are expressed in terms of fold changes of β-actin. The expression of pro-BDNF, but not m-BDNF, was upregulated before MCAO, while p75NTR expression was significantly reduced both before and after MCAO. The results are expressed as the mean ± standard error of the mean. *p < 0.05, **p < 0.01, ***p < 0.001.

To investigate how sEH is involved in postsynaptic excitability, we then investigated BDNF-TrkB and p75 neurotrophin receptor (p75NTR) signaling, which induces NMDAR trafficking and activation. Compared to vehicle treatment, AUDA treatment upregulated mature BDNF (17 kDa) and phosphorylated TrkB (p-TrkB) (90 kDa, phosphorylated Y515). Pro-BDNF (35 kDa) and p75NTR (70 kDa) levels did not differ between groups (Fig. 3A). On the other hand, the sEH KO mice exhibited overexpression of pro-BDNF and p-TrkB, but not BDNF, at baseline and persistently upregulated p-TrkB after MCAO compared to the WT group. In the sEH KO mice, p75NTR expression was downregulated at the normal state and after MCAO (Fig. 3B). Enhancement of TrkB phosphorylation and downregulation of p75NTR suggests that these effects might be mediated more by BDNF-TrkB than by pro-BDNF-p75NTR signaling, which may enhance neuronal survival^34^ instead of apoptosis^35^. Taken together, sEH inhibition and deletion consistently increased the activation of the neurotrophin TrkB, which may mediate the amelioration of infarcts and neurological deficits. The student’s t-test was used to determine the significance of the differences in protein levels.

### Pharmacological inhibition and sEH gene deletion increase neuronal TrkB and microvasculature in the peri-infarct cortex

**Figure 4:**
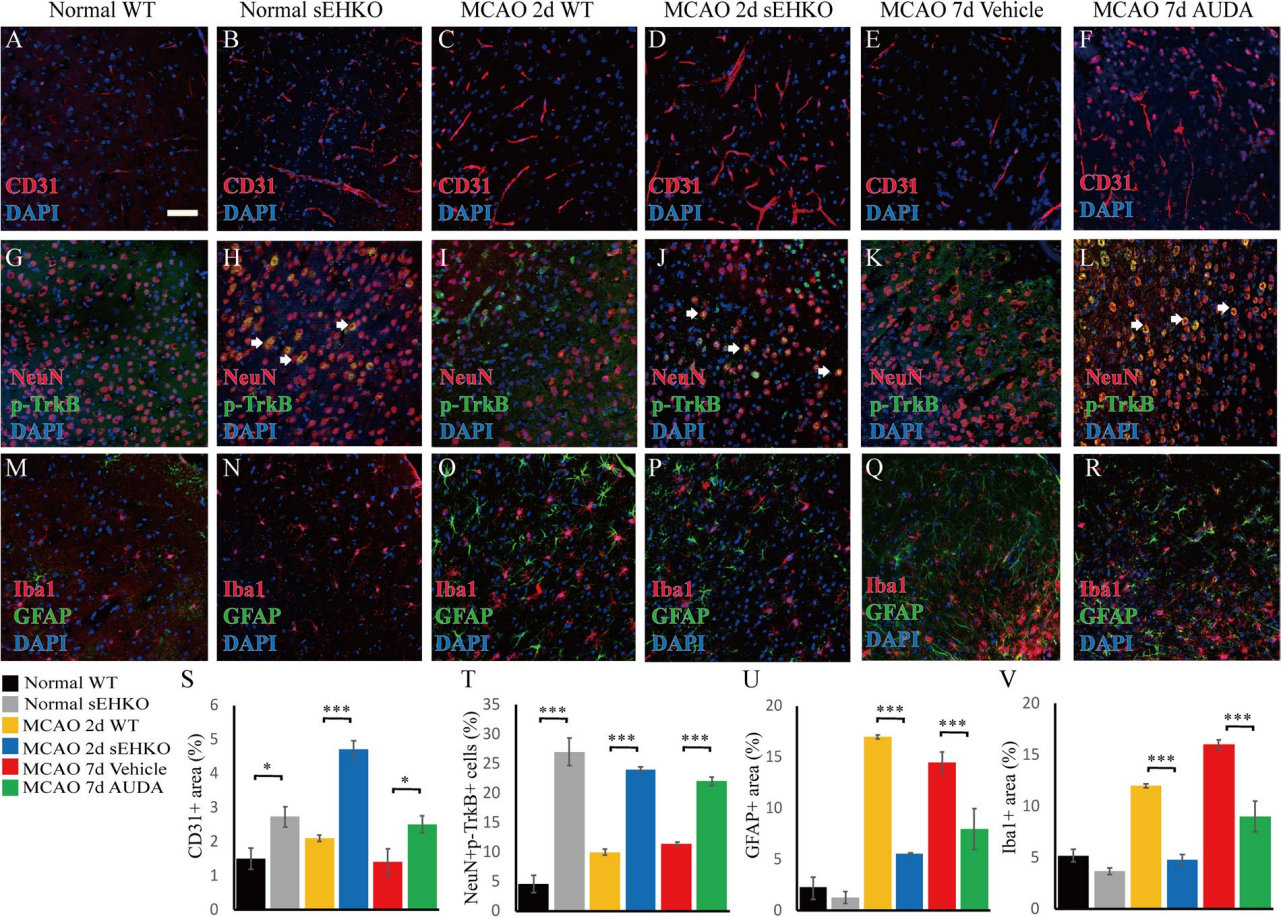
Representative and quantitative immunohistochemistry of the peri-infarct cortex before the bregma (coordinate + 0.58) in normal WT, normal sEH KO, MCAO WT, MCAO sEH KO, MCAO with vehicle treatment and MCAO with AUDA treatment mice (n = 2 for normal groups, n = 5–6 for all groups subjected to MCAO). (**A**–**F**, **S**) The number of CD31-positive endothelial cells significantly increased in the sEH KO before and after MCAO and post-MCAO AUDA-treated mice, while (**G**–**L**) Note the increased phosphorylated TrkB (p-TrkB) immunoreactivity that partially co-localized with NeuN-positive neurons, particularly in the sEH KO and AUDA-treated mice (arrows) (**T**). The percentage of NeuN- and p-TrkB-positive double labeling in the neuronal population was significantly increased compared to that in the corresponding controls. (**M**–**R**, **U**–**V**) GFAP-positive astrogliosis and Iba1-positive microglial infiltration decreased in post-MCAO sEH KO and AUDA treatment groups relative to that in the corresponding controls. The scale bar represents 50 µm. *p < 0.05, **p < 0.01, ***p < 0.001.

Pharmacological inhibition of sEH significantly increased CD31-positive endothelial cells in the peri-infarct region, while increased CD31-positive cells were observed before and after MCAO in sEH KO mice (Fig. 4A–F,S). Additionally, more NeuN-positive neuronal populations that were double-labeled for p-TrkB immunoreactivities were observed in the AUDA-treated group compared to the vehicle control group, while the sEH KO group exhibited sustained increases in p-TrkB before and after MCAO (Fig. 4G–L,T). These findings again suggest differences between the transient nature of pharmacological inhibition and the permanent gene deletion followed by adaptations in knockouts. Furthermore, decreased GFAP-positive astrogliosis and decreased Iba1-positive microglial infiltration by sEH inhibition and deletion (Fig. 4M–R,U–V) were found in the peri-infarct cortex, although the decreases did not differ between the normal WT and sEH KO groups, consistent with previous reports in experimental stroke^36^. Nevertheless, other studies of heart and kidney injury reported that in contrast to pharmacological inhibition, sEH gene deletion aggravated myocardial fibrosis and renal tubular inflammation in association with elevated HETEs, which might have an effect opposite to that of EETs on vascular regulation and inflammation^27,28^. However, such pro-inflammatory effects of HETEs were not observed here in sEH KO before or after stroke. The cerebral microvasculature of sEH KO was more abundant than that of WT mice regardless of elevated 20-HETE levels. This may be due to complex substrate shifting in transgenic mice after generations of adaptation to the changes caused by lack of sEH. Collectively, these findings suggest that increased p-TrkB in neurons, as well as enhanced collateral microvasculature and reduced inflammatory gliosis, may be associated with the neuroprotection mechanisms by sEH blockade. The student’s t-test was used to determine the significance of differences in quantitative immunoreactivities.

### sEH blockade-mediated neuroprotection requires TrkB overactivation

**Figure 5:**
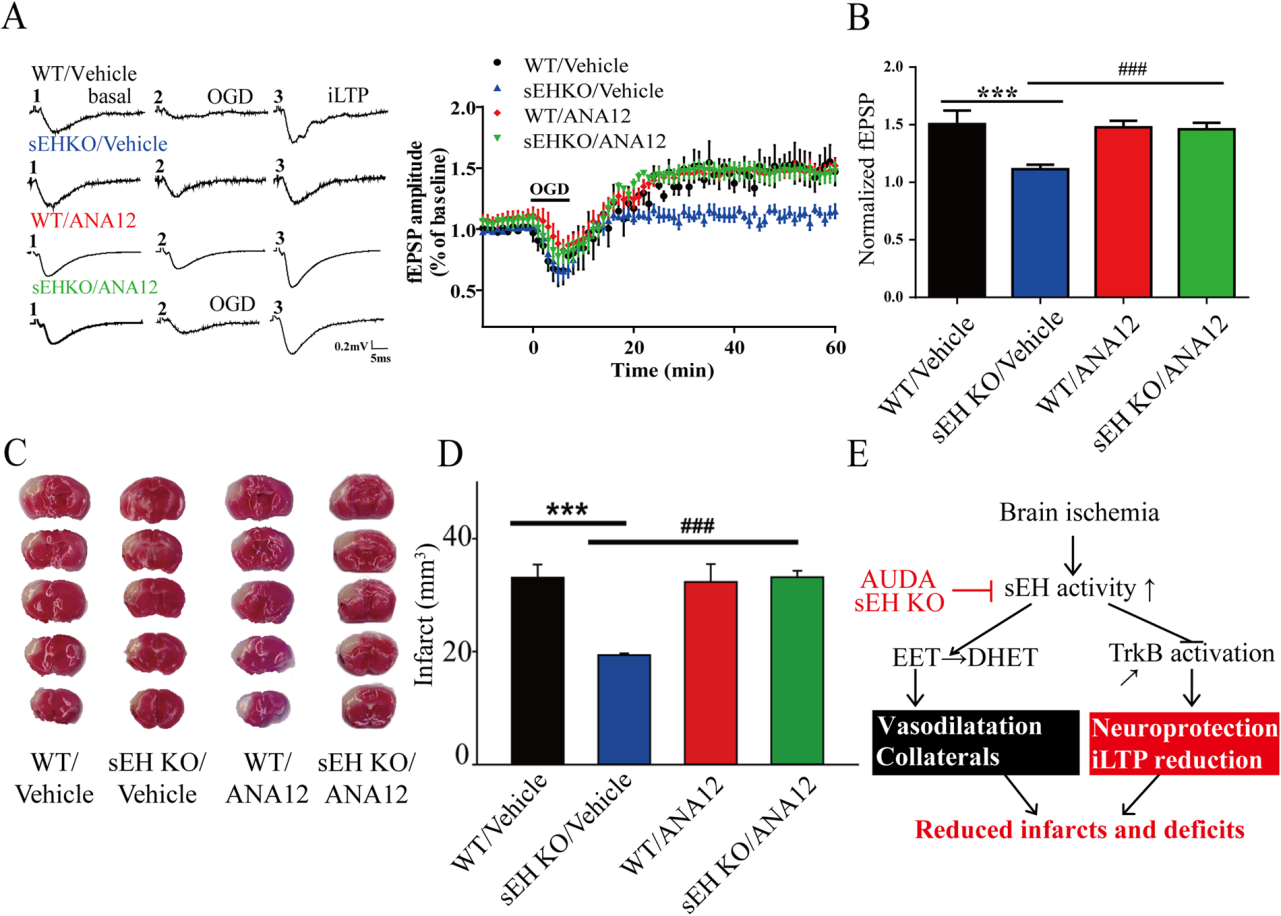
The functional relevance of TrkB overactivation in the sEH knockout (sEH KO) mice evaluated by pretreatment with the selective TrkB antagonist ANA12 before oxygen-glucose deprivation (OGD) *ex vivo* and before middle cerebral artery occlusion (MCAO) *in vivo*. (**A**) Representative traces of the field excitatory postsynaptic potentials (fEPSPs) in the hippocampus at different time points at baseline, during OGD induction and during post-ischemic long-term potentiation (iLTP) expression. The averaged time course of fEPSP changes over 60 minutes following OGD induction is shown for WT and sEH KO mice with either vehicle or ANA12 pretreatment. (**B**) During iLTP expression in the last 10 minutes, the average fEPSP amplitude significantly decreased in the sEH KO/vehicle group relative to the WT/vehicle group; this effect was abolished by ANA12 pretreatment in the sEH KO group (sEH KO/ANA12, n = 6/group). (**C**) Vital staining of representative brain slices at 48 hours after MCAO from WT and sEH KO mice with either vehicle or ANA12 pretreatment. (**D**) ANA12 pretreatment (sEH KO/ANA12, n = 6) significantly eliminated the infarct reduction in the sEH KO/vehicle relative to the WT/vehicle (n = 6/group), suggesting that the protective effects of sEH deletion on the attenuation of iLTP and infarction were mediated by TrkB activation. (**E**) A mechanistic framework summarizing the effects of sEH blockade. Blocking sEH induces TrkB overactivation and prevents EET degradation, leading to neuroprotection, iLTP reduction, vasodilatation, and, finally, reductions in infarcts and their associated deficits. Calibration: 0.2 mV, 5 ms. The values represent the mean ± standard error of the mean. *,^#^ *p* < 0.05, ***,^###^ *p* < 0.001.

To determine whether TrkB mediates the neuroprotection conferred by sEH blockade and deletion, ANA12, a selective TrkB receptor inhibitor, was used for pretreatment in the OGD and MCAO models. During OGD induction, the fEPSP amplitude did not differ among the WT/vehicle, WT/ANA12, sEH KO/vehicle and sEH KO/ANA12 groups (Fig. 5A). During iLTP expression, we found that the iLTP amplitude of the WT/vehicle and WT/ANA12 groups increased similarly, suggesting that ANA12 did not affect injury (Fig. 5A,B). Importantly, the iLTP amplitude of the sEH KO/ANA12 group was significantly higher than that of the sEH KO/vehicle group and similar to that of the WT/vehicle group, suggesting that sEH KO reduced iLTP through TrkB signaling (Fig. 5A,B). Similarly, we found that WT/ANA12 pretreatment did not affect the brain infarct compared to that observed in the WT/vehicle controls; however, sEH KO/ANA12 reversed the protective effects of sEH KO/vehicle *in vivo* 48 hours after MCAO (Fig. 5C,D). Together, the findings suggest a novel framework for how sEH increases after ischemia potentiate injury through negative regulation of TrkB and EET levels (Fig. 5E). Hence, AUDA inhibition and sEH gene deletion not only increased EET levels to enhance cerebrovascular collaterals but epoxide fatty acids might possibly activate TrkB to protect neurons and reduce iLTP hyperexcitability, leading to decreased infarcts and functional deficits. The student’s t-test was used to determine the statistical significance of differences in fEPSP amplitude and infarct volume.

## Discussion

Both pharmacological inhibition (AUDA) and genetic deletion of sEH significantly ameliorated ischemic infarction and sensorimotor deficits in association with overexpression of brain p-TrkB and NMDA-type glutamate receptors in adult mice. Moreover, neuronal expression of p-TrkB, as well as collateral microvasculature, were significantly enhanced in the peri-infarct cortex, while astrogliosis and microglial activation were reduced. We demonstrated, for the first time, that both the inhibition and deletion of sEH reduced post-ischemic neuronal hyperexcitability, i.e., iLTP, in an *ex vivo* OGD model of adult hippocampal slices. Importantly, pretreatment with a selective TrkB inhibitor before OGD and before MCAO reversed the iLTP and infarct attenuation conferred by sEH deletion, suggesting that TrkB activation was involved in the neuroprotective effects. Together, these findings indicate that sEH potentiates ischemic injuries and neuronal hyperexcitability not only through the degradation of protective EETs but also through negative regulation of the TrkB activation.

The impact of sEH on synaptic plasticity is largely unknown. We previously reported that sEH inhibition (AUDA) enhanced neuronal synaptic transmission and physiological long-term potentiation (LTP) following high-frequency stimulation using whole-cell and extracellular recordings in the mouse prefrontal cortex *ex vivo*. These enhancements were associated with the overexpression of postsynaptic NMDAR and AMPAR subunitsas well as extracellular signal-regulated kinase (ERK) phosphorylation^37^. The activation of ERK signaling mediates neuronal responses to neurotrophic factors and is required for NMDA-dependent LTP^38^. By contrast, here we demonstrate that both sEH inhibition and gene deletion decreased iLTP following OGD insult using extracellular recordings in the hippocampus. These decreases were associated with the overexpression of p-TrkB; however, the upregulation of NMDAR and AMPAR subunits was inconsistent across the models. The addition of the selective TrkB inhibitor ANA12 reversed the attenuation of iLTP *ex vivo* and the infarct size reduction *in vivo*. Hence, the neuroprotective effect of sEH inhibition may be mediated by TrkB activation rather than through glutamate receptors. It has been well documented that BDNF-TrkB activation enhances synaptic transmission and plasticity^21,39,40^. Recently, it has been suggested that sEH deletion and 14,15-EETs promoted astrocyte-derived BDNF production and exerted neuroprotective effects during OGD injury through BDNF-TrkB-ERK activation *in vitro* ^18^. Moreover, pharmacological inhibition and genetic deletion of sEH have been shown to prevent the onset of depression-like behaviors induced by repeated experimental social defeat stress in association with upregulated BDNF-TrkB expression in the prefrontal cortex and hippocampus^19^. Our findings suggest that sEH blockade may not only enhance physiological LTP but also attenuate OGD-induced iLTP. The attenuation of iLTP and infarction against ischemic injury by pharmacological inhibition and genetic deletion of sEH involves, at least in part, TrkB activation. The neural mechanisms can be further elucidated using forebrain-specific^41,42^, glia-specific or neuron-specific^43^ TrkB knockout mice.

There are limitations to our study, and further studies are needed. First, we did not have direct 14,15-EET measurement data because a liquid chromatograph-mass spectrometer was not available, and the manufacturer of the ELISA kit stopped production before we could finish our experiments. According to previous publications, sEH blockade likely increases EET levels in the brain^26^. It is unknown whether the neuroprotective effects of iLTP attenuation and p-TrkB overexpression might be EET-dependent. Second, we could not determine which glutamate receptor might mediate iLTP modulation via sEH blockade using extracellular fEPSPs. Whole-cell patch clamp recordings or synaptoneurosome isolation would be required to make this distinction^44^. The mechanisms underlying the effects of sEH on synaptic plasticity remain unclear. Third, we can not exclude possible vascular mechanisms by sEH blockade because AUDA treatment, but not sEH KO, lowered blood pressure after MCAO. Nevertheless, we did not observe significant changes in peri-infarct blood flow by sEH blockade. Fourth, we used an *ex vivo* hippocampal OGD model for convenience of iLTP recording, which is not supplied by MCA, therefore not accurately reflective of an *in vivo* MCAO model. Fifth, BDNF mRNA and protein are present at extremely low levels in the brain^20^, and after ischemic injury, brain BDNF expression increases in a transient manner and then dramatically decreases^45^. Consistent with TrkB overexpression already before MCAO, sEH gene deletion did not significantly increase BDNF protein levels after MCAO in the current study. Additionally, we could not completely rule out that neurotrophin4-TrkB signaling also plays a role in TrkB activation. Finally, the pharmacokinetics of ANA12 *in vivo* limit its effects to 4 hours^23^; more frequent administration of ANA12 may be feasible for TrkB inhibition.

In conclusion, we demonstrated the protective potential of sEH inhibition after acute ischemic stroke in amelioration of functional deficits and provided evidence that this protection involves TrkB activation. Pharmacological inhibition and gene deletion of sEH attenuated iLTP following OGD insult in the hippocampus *ex vivo*, and this effect was reversed by selective TrkB inhibition. sEH genetic polymorphisms may increase or decrease sEH activity^46^, which might affect TrkB activity. Our findings provide novel insights into the TrkB-mediated neuroprotection and ischemic resiliance conferred by pharmacological inhibition and gene deletion of sEH.

## Materials and Methods

The datasets generated or analyzed during the current study are available from the corresponding author on reasonable request.

### Permanent MCAO model and study design

Adult male C57BL/6 J WT mice (10–12 weeks old, 22–26 g body weight, BioLASCO Taiwan Co., Ltd, n = 123) and sEH KO mice (B6.129X-*Ephx2* ^*tm1Gonz*^/J, The Jackson Laboratory, n = 53) were anesthetized with intraperitoneally administered chloral hydrate (450 mg/kg body weight in saline) and received permanent middle cerebral artery occlusion (MCAO) surgery using a procedure described previously^47^. Briefly, the dominant limb was determined based on the skilled forelimb reaching test (see below). Subsequently, the distal MCA contralateral to the dominant limb was exposed by craniotomy and directly cauterized, followed by simultaneous occlusion of the bilateral common carotid arteries using microclips for 20 minutes to paralyze the dominant forelimb. The body temperature was maintained with a homeothermic heating pad with rectal temperature control at 37 ± 0.5 °C. For the pharmacological inhibition of sEH, 61 WT mice were randomly allocated to receive a daily intraperitoneal injection of either vehicle (2% dimethyl sulfoxide (DMSO), Sigma-Aldrich, n = 40) or AUDA^48^ (10 mg/kg in 2% DMSO, Cayman Chemical, n = 41) for 7 consecutive days post-MCAO. The animal experiments were approved by the Institutional Animal Care and Use Committee at the Taipei Veterans General Hospital. All methods were performed in accordance with the relevant guidelines and regulations.

### Behavioral tests

Before MCAO and 1 to 7 days after, blind observers longitudinally measured performance on the rotarod test^49,50^, the skilled forelimb reaching task (pasta matrix reaching task)^51^ and the adhesive removal test^52^ (AUDA n = 9 vs. vehicle n = 10; sEH KO n = 5 vs. WT n = 6). For the rotarod test, the mice were pre-trained for 5 days before surgery. Within each session, the rotarod speed was increased from 4 to 40 rpm with an acceleration rate of 8 rpm/min. The mice performed 3 times of 10-minute test sessions of running. The latency to fall from the rotarod was recorded and averaged. For the skilled forelimb reaching task, i.e., the pasta matrix reaching task, the mice were pre-trained for 2 weeks to learn to break and retrieve pieces of uncooked capellini from a row of vertically oriented pieces of pasta (matrix) until they achieved a maximum of 30 pieces in 30 minutes. The mouse was placed in an acrylic chamber (15 × 8.5 × 20 cm) containing a slit (13 × 0.5 cm at the center of the 15 cm long side). The capellini matrix was then placed in front of the slit. The number of pieces of pasta removed in 30 minutes were recorded for analysis. For the adhesive removal test, a forelimb was cleaned, and a piece of adhesive tape (0.3 × 0.4 cm) was attached to the limb. The timer started once the mice were placed into a cylinder. The time at which the mouse began to remove the tape and the time at which the tape was completely removed was recorded.

### Quantification of infarcts, sEH activity, fatty acids and western blotting

After 2 or 7 days of MCAO and behavioral measurements, the mice were decapitated after intraperitoneal injection of urethane (1–1.5 g/kg). Immediately after, fresh brains were harvested for quantification of infarct size, sEH activity, fatty acids, and proteins. To quantify the infarct, we sectioned the brain into standard 1 mm coronal slices using a brain matrix slicer (Jacobowitz Systems, Zivic-Miller Laboratories Inc.), stained the brain slices with a 2% solution of the vital dye 2,3,5-triphenyltetrazolium chloride (TTC, Sigma-Aldrich) at 37 °C for 30 minutes in the dark and fixed them with 10% formalin at room temperature overnight (AUDA n = 18 vs. vehicle n = 17; sEH KO n = 8 vs. WT n = 8). The area of the infarct was delineated and measured using AIS software (Imaging Research Inc.), and the total volume (in mm^3^) was calculated as the sum of the infarct areas on each slice^53^. Both the surgeon and analyst were blind to the experimental groups.

To measure sEH activity and fatty acids, the ipsilesional hemisphere was isolated, weighed and homogenized with additional T-PER Tissue Protein Extraction (5 µl/mg tissue, ThermoFisher) (n = 5/group). The hydrolase activity of sEH was determined using a cell-based assay kit (Cayman Chemical)^2,54^, while levels of 14,15-DHET and 20- HETE were determined using enzyme-linked immunosorbent assay (ELISA) kits immediately after the tissue was homogenized according to the manufacturer’s instructions (Detroit R&D). For DHET ELISA, the homogenate was acidified with acetic acid, extracted with an equal amount of ethyl acetate several times and then dried using a vacuum concentrator (ThermoFisher). The residue was dissolved in ethanol and diluted with dilution buffer for the ELISA.

For western blotting, the aforementioned fresh homogenate that was prepared in T-PER Tissue Protein Extraction buffer (ThermoFisher) was mixed with 0.5% protease inhibitor (Sigma-Aldrich) and 0.5% phosphatase inhibitor cocktail 2 and 3 (Sigma-Aldrich) and centrifuged at 10000xg for 20 minutes to obtain supernatants (n = 8/group). The protein concentration of the supernatants was determined using a NanoVue Plus Spectrophotometer. Equal amounts of protein (40 µg) were loaded onto 7.5%, 12% or 15% gels depending on the target protein size. The proteins were then separated using SDS-PAGE, transferred onto polyvinylidene difluoride membranes and blocked with 5% skim milk powder (Fluka) for 60 minutes. The following primary antibodies were then used for incubation overnight at 4 °C: rabbit anti-BDNF (1:500, Origene, TA328615), rabbit anti-phosphorylated TrkB (p-TrkB, 1:500, Abcam, ab109684), rabbit anti-GluN2A (1:1000, Millipore, 05–901 R), rabbit anti-GluN2B (1:1000, Cell Signaling, 4207 S), rabbit anti-p75NTR (1:1000, Abcam, ab52987), rabbit anti-GluA2 (1:1000, Origene, TA326000) and rabbit anti-β-Actin (1:2000, ThermoFisher, PA1-183). The membranes were then incubated with secondary HRP-conjugated antibodies (1:5000, Millipore, AP132P) for 1 hour at room temperature. The immunoreactivity was detected with Clarity Western ECL Substrate (Bio-Rad). X-ray films (Fuji) were exposed for the different duration to ensure optimal densities. Relative optical densities were normalized to the levels of the internal control β-actin and then measured as fold changes using ImageJ^55^.

### Immunohistochemistry

After 2 or 7 days of MCAO, the mouse was anesthetized and transcardially perfused with 20 ml normal saline containing 10 U/ml heparin followed by 20 ml 4% paraformaldehyde in phosphate-buffered saline (PBS) (AUDA vs. vehicle n = 5/group; sEH KO vs. WT n = 6/group). The brain was dissected and post-fixed in 4% paraformaldehyde overnight and then transferred to 30% sucrose for days. Afterward, 20 µm thickness frozen cryostat brain sections were attached to silane-coated glass slides. The slides were incubated in a blocking solution containing 3% donkey serum albumin (Abcam) and 0.3% Triton X-100 (Sigma-Aldrich) in TBS for 1 hour at room temperature and immunostained with primary antibodies including rabbit anti-CD31 (1:200, BioRad, MCA23886A), anti-p-TrkB (1:200, Abcam, ab109684), anti-NeuN (1:200, Abcam, ab104224), anti-GFAP (1:500, Abcam, ab7260), and anti-Iba1 (1:200, Novus, NB100-1028) overnight at 4 °C. The slides were then further incubated with fluorescence marker-conjugated secondary antibodies (Alexa 488 and Cy3, all donkey host, 1:200, Jackson immune research) for 2 hours at room temperature in the dark. After the slides were washed thoroughly with TBS, coverslips were sealed onto the glass slides with Vectashield Fluorescent Mounting Medium containing DAPI nuclear counterstain (Vector Laboratories). Images were acquired and averaged over a standardized sampling sites of 3 slices per mouse and 2–6 mice per group (n = 2 for two normal groups, n = 6 for MCAO 2 d WT, n = 6 for MCAO 2 d sEH KO, n = 5 for MCAO 7 d Vehicle, n = 5 for MCAO 7 d AUDA) using an Olympus FV1000i confocal laser scanning microscope. Quantification was performed with ImageJ^55^.

### Electrophysiological recording of hippocampal slices, oxygen-glucose deprivation, and iLTP induction

Adult WT (n = 12) and sEH KO (n = 18) mice were decapitated, and their brains were immediately dissected and placed in ice-cold (4 °C) oxygen-saturated (95% O_2_ and 5% CO_2_) artificial cerebrospinal fluid (ACSF, pH = 7.4). The brain slices (400 µm thickness) were prepared as described previously^56^ and placed in a beaker of oxygenated ACSF at room temperature for at least 1 hour before recording. A single slice was transferred to a recording chamber consisting of a circular well (2 ml volume) and perfused constantly at a rate of 2–3 ml/min at 32 ± 1 °C. A bipolar stimulating electrode (FHC, Bowdoin) was inserted into the Schaffer collaterals to deliver electrical stimuli (duration 150 µs; frequency 0.1 Hz). Field excitatory postsynaptic potentials (fEPSPs) were recorded from the stratum radiatum of the CA1 subregion with a glass pipette containing 1 M NaCl and a Multiclamp 700B amplifier. Baseline field potentials were recorded every 10 s for 10 minutes and adjusted to 30–40% of the maximal responses. For OGD and iLTP induction, glucose-containing ACSF gassed with 95% O_2_/5% CO_2_ was replaced with sucrose-containing ACSF gassed with 95% N2/5% CO2 for 5 minutes. Then, the perfusion solution was switched back to normal ACSF, and the slices were re-oxygenated with 95% O_2_/5% CO_2_^57,58^. iLTP was continuously recorded for 60 minutes after OGD. The average fEPSP amplitude during the 5 minutes of OGD induction and the last 10 minutes of iLTP expression were compared across groups (n = 6 slices/group).

### Selective TrkB blockade *in vivo* and *ex vivo*

To investigate whether TrkB mediated the effects of sEH inhibition and deletion on the attenuation of brain infarction and iLTP, we pre-treated the experimental groups with the selective TrkB inhibitor ANA12, before MCAO and OGD, respectively. WT and sEH KO mice were pretreated with intraperitoneal injection of ANA12 (0.5 mg/kg, MedKoo; n = 5/group) or vehicle (1% DMSO, Sigma-Aldrich; n = 3/group) every 12 hours for 3 times before the MCAO surgery to ensure TrkB inhibition. For the *ex vivo* experiments, the hippocampal slices from WT and sEH KO mice were pretreated with 10 µM ANA12 or vehicle for 30 minutes before OGD (n = 6 slices/group).

### Statistical Analysis

We used SPSS (Windows version 22) for the data analyses. The repeated-measures analysis of variance test (ANOVA) was used to compare the behavioral data, with “time point” as the within-subject factor and “group” as the between-subjects factor. The ANOVA was followed by post-hoc Bonferroni tests at every time point to determine any group difference. We used independent Student’s t-tests or one-way ANOVA to evaluate group differences in infarct size, sEH activity, lipid metabolites, western blotting, immunohistochemistry and fEPSP amplitude measurements. A *p*-value < 0.05 was considered statistically significant.

## Electronic supplementary material

Supplementary Information
