# Supplementary material for: Blockade of soluble epoxide hydrolase attenuates post-ischemic neuronal hyperexcitation and confers resilience against stroke with TrkB activation
Source: Sci Rep. 2018 Jan 8;8:118. doi: 10.1038/s41598-017-18558-6 (PMC5758800; doi:10.1038/s41598-017-18558-6)
Supplement: Supplementary file 1 — Supplementary Information [file 41598_2017_18558_MOESM1_ESM.doc]

***Supplementary Information***

**Blockade of Soluble Epoxide Hydrolase Attenuates Post-ischemic Neuronal Hyperexcitation and Confers Resilience Against Stroke with TrkB Activation**

Li-Hsin Chang^1^ BS; Hui-Ching Lin^2^ Ph.D.; Shiang-Suo Huang^3^ Ph.D.; I-Chih Chen^1^ MS; Kai-Wen Chu^2^ MS; Chun-Lien Chih^4^ AS; Yao-Wen Liang^5^; Yi-Chung Lee^6,7^ M.D., Ph.D.; You-Yin Chen^8^ Ph.D.; Yi-Hsuan Lee^2^ Ph.D.; I-Hui Lee^1, 6*^ M.D., Ph.D.

^1^Institute of Brain Science, National Yang-Ming University, Taipei, Taiwan; ^2^Department and Institute of Physiology, National Yang-Ming University, Taipei, Taiwan; ^3^Department of Pharmacology, Institute of Medicine, Chung-Shan Medical University, Taichung, Taiwan; ^4^Cheng Hsin General Hospital, Taipei, Taiwan; ^5^Department of Life Sciences and Institute of Genome Sciences, National Yang-Ming University, Taipei, Taiwan; ^6^Department of Neurology, Taipei Veterans General Hospital, Taipei, Taiwan; ^7^Department of Neurology, School of Medicine National Yang-Ming University, Taipei, Taiwan; ^8^Department of BioMedical Engineering, National Yang-Ming University, Taipei, Taiwan

***Corresponding author**: I-Hui Lee, M.D., Ph.D.

Address: No. 201, Sec. 2, Shipai Rd., Beitou District, Taipei City, Taiwan 11217

Phone: +886-2-28712121 ext. 8109

Fax: +886-2-28757579

E-mail address: [ihlee@vghtpe.gov.tw](mailto:ihlee@vghtpe.gov.tw)

**Supplementary methods**

**Blood pressure measurement**

Arterial blood pressure was recorded in mice using a non-invasive blood pressure monitor (Model MK-2000ST, Muromachi) in which the cuff was placed at the base of the tail to investigate the vascular effects of pharmacological inhibition and genetic deletion of soluble epoxide hydrolase (sEH). We longitudinally compared the mean blood pressure of WT/vehicle (n=4), WT/AUDA (n=4), and sEH KO (n=5) mice at 3 time points after anesthesia according to the manufacturer’s instructions: before MCAO, 15 minutes after reperfusion (and vehicle or AUDA treatment), and 24 hours after MCAO. The statistical analysis used to compare the data among groups was one-way ANOVA followed by a post-hoc Bonferroni test.

**Quantitative measurement of cortical collateral blood flow**

In the above mice, we also used an in-house laser speckle contrast imaging system^1,2^ to longitudinally measure cerebral blood flow at a high temporospatial resolution, as previously reported^3^. Briefly, anesthetized mice were fixed in a stereotaxic instrument to prevent head movements and a craniotomy was performed over the distal middle cerebral artery (MCA). Reflectance surface images of the cortical region of interest (3 mm x 3 mm) were generated with a continuous-wave 100 mW 532 nm laser module (GM-XP02-100, Unice E-O Service Inc.), and integrated speckle images were generated with a 100 mW 660 nm laser module (RM-CW04-100, Unice E-O Service Inc.). All images were recorded with a 16-bit charge-couple device camera (Point Grey Research Inc., DR2-08S2M/C-EX-CS) at a resolution of 1032x776 pixels and at a rate of 15 frames per second for 2 minutes per time point. We compared laser speckle images obtained from vehicle-treated WT (n=4), AUDA-treated WT (n=4), and sEH KO (n=5) mice at 4 time points: before MCAO, immediately after MCAO, 15 minutes after reperfusion (and vehicle or AUDA treatment), and 24 hours after MCAO. Post-ischemic changes were expressed relative to the baseline blood flow before MCAO to adjust for individual differences in cerebral blood flow. Relative cerebral blood flow was analyzed using MATLAB R2016b spm12 toolbox to locate the cortical vasculature and calibrate the vibration. Repeated measures ANOVA was applied to compare data among groups.


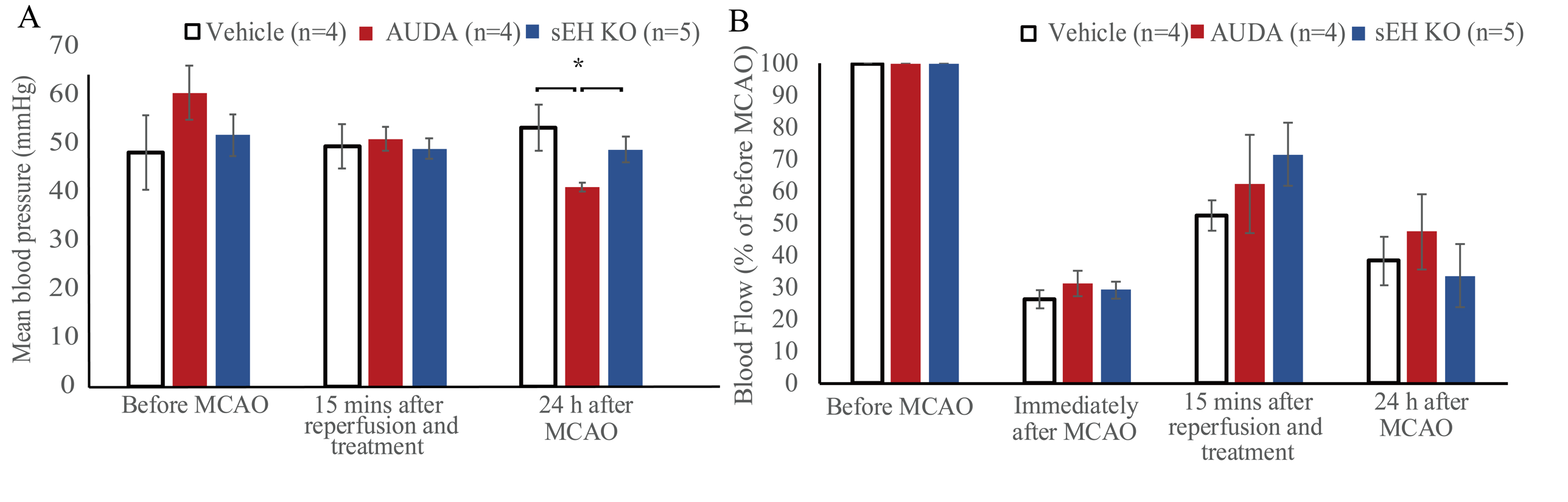


**Supplementary Figure 1.** (A) Longitudinal changes of blood pressure in the vehicle-treated, AUDA-treated, and soluble epoxide hydrolase knockout (sEH KO) groups. Notably, the AUDA-treated group displayed a significant decrease in mean blood pressure 24 hours after middle cerebral artery occlusion (MCAO) compared with the vehicle-treated and sEH KO groups. (B) Simultaneous changes in the peri-infarct cortical blood flow rate relative to the baseline rate before MCAO. Drastic reductions in cortical blood flow were observed immediately after MCAO in the 3 groups compared to the baseline values. However, no significant difference was observed among the groups by 24 hours after MCAO. The results are expressed as means ± standard errors of the means. **p*<0.05.

**
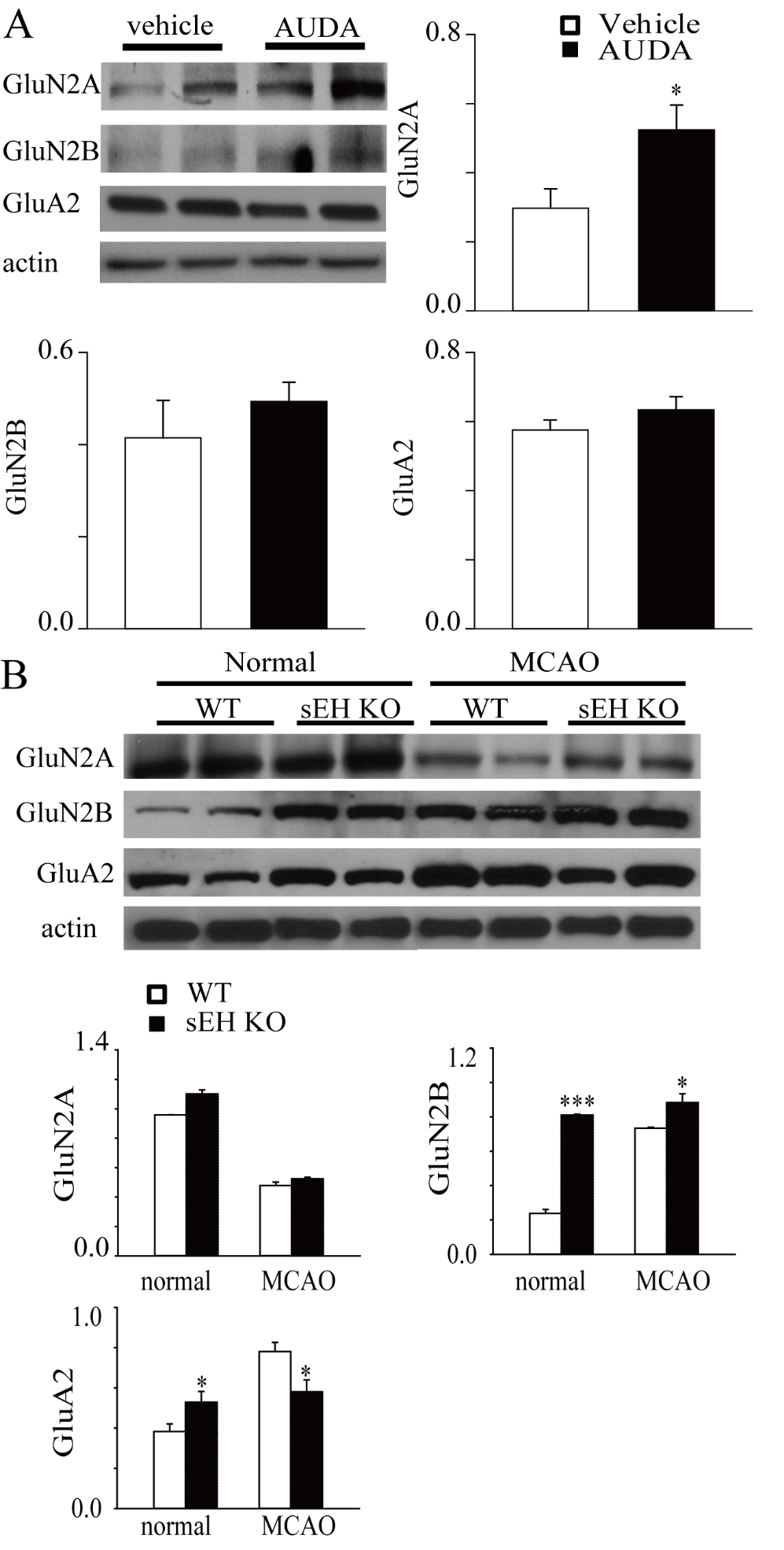
**

**Supplementary Figure 2.** (A) Representative western blots of the ipsilesional hemisphere from vehicle- and AUDA-treated mice, with β-actin as the loading control. Following 7 days of treatment after MCAO, the AUDA treatment (10 mg/kg/day) significantly increased the level of the GluN2A subunit of the NMDA receptor proteins, but did not change the level of GluN2B or GluA2 subunits of the AMPA receptors compared to the vehicle treatment (n=8 mice/group). (B) Representative western blots of wild-type (WT) and sEH knockout (sEH KO) mice before (normal) and 48 hours after MCAO. Levels of GluN2A subunit proteins were not different between the WT and sEH KO mice before or after MCAO. In contrast, the level of GluN2B subunits was increased in sEH KO mice before and after MCAO. Additionally, the GluA2 level was substantially increased in sEH KO mice before MCAO but was decreased after MCAO. The results are expressed as means ± standard errors of the means. **p*<0.05, ****p*<0.001.


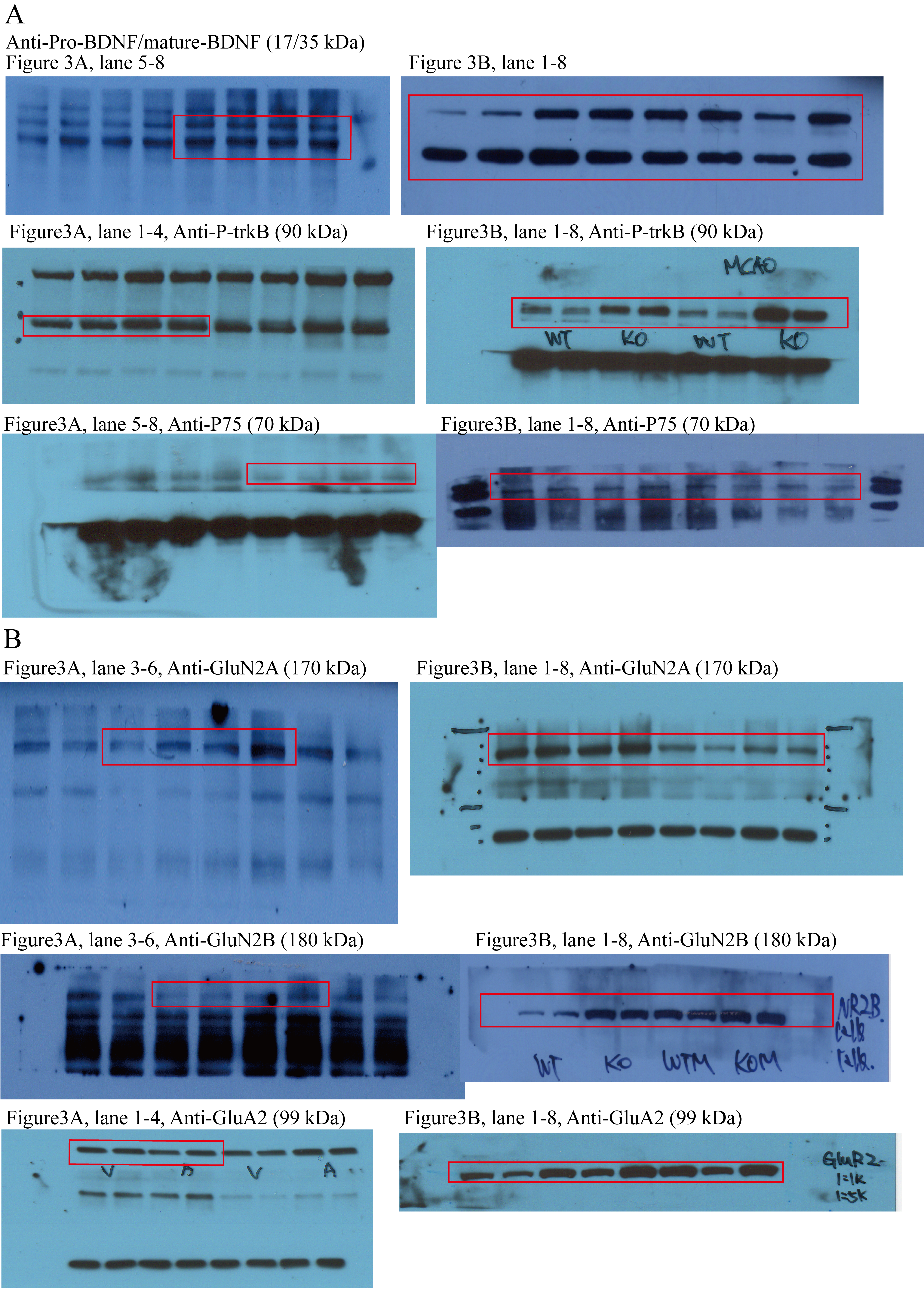


**Supplementary Figure 3.** Uncropped images for western blots and SDS-PAGE. Red box marks the borders of the final cropped image. (A) Original pictures of Figure 3. (B) Original pictures of supplementary Figure 2.

References

1 Boas, D. A. & Dunn, A. K. Laser speckle contrast imaging in biomedical optics. *Journal of Biomedical Optics* **15**, 011109 (2010).

2 Senarathna, J., Rege, A., Li, N. & Thakor, N. V. Laser Speckle Contrast Imaging: theory, instrumentation and applications. *IEEE reviews in biomedical engineering* **6**, 99-110 (2013).

3 Pan, H.-C. *et al.* Neurovascular function recovery after focal ischemic stroke by enhancing cerebral collateral circulation via peripheral stimulation-mediated interarterial anastomosis. *Neurophotonics* **4**, 035003 (2017).
